# Novel Superhydrophobic Surface with Solar-Absorptive Material for Improved De-Icing Performance

**DOI:** 10.3390/ma12172758

**Published:** 2019-08-28

**Authors:** Joseph Gonzales, Daiki Kurihara, Tetsuro Maeda, Masafumi Yamazaki, Takahito Saruhashi, Shigeo Kimura, Hirotaka Sakaue

**Affiliations:** 1 Department of Aerospace and Mechanical Engineering, University of Notre Dame, White Field Research Laboratory, Notre Dame, IN 46556, USA; 2 Department of Mechanical Engineering Kanagawa Institute of Technology, Atsugi, Kanagawa 243-0292, Japan

**Keywords:** superhydrophobic coating, thermal management

## Abstract

Ice accretion is detrimental to numerous industries, including infrastructure, power generation, and aviation applications. Currently, some of the leading de-icing technologies utilize a heating source coupled with a superhydrophobic surface. This superhydrophobic surface reduces the power consumption by the heating element. Further power consumption reduction in these systems can be achieved through an increase in passive heat generation through absorption of solar radiation. In this work, a superhydrophobic surface with increased solar radiation absorption is proposed and characterized. An existing icephobic surface based on a polytetrafluoroethylene (PTFE) microstructure was modified through the addition of graphite microparticles. The proposed surface maintains hydrophobic performance nearly identical to the original superhydrophobic coating as demonstrated by contact and roll-off angles within 2.5% of the original. The proposed graphite coating also has an absorptivity coefficient under exposure to solar radiation 35% greater than typical PTFE-based coatings. The proposed coating was subsequently tested in an icing wind tunnel, and showed an 8.5% and 50% decrease in melting time for rime and glaze ice conditions, respectively.

## 1. Introduction

Ice accumulation causes irreversible damage in systems ranging from ships, heat pumps, civil infrastructure, and aircraft [1,2,3,4,5]. The negative impact of ice formation can manifest itself both in structural damage, as well as in reduction of efficiency, which can lead to total system failure. Ice formation in aircraft and wind turbine applications has been shown to reduce lift and cause in-flight burnout [6]. In order to tackle many of these issues, there are a variety of systems which prevent ice formation—anti-icing systems—and remove accumulated ice—de-icing systems—that are the subject of ongoing research.

The most common of these systems attempt to reduce ice adhesion and accumulation using mechanical and electrothermal techniques [1,7]. Such applications by their very nature are often energetically expensive, heavy, or difficult to implement. Especially for power generation and aerospace applications, these drawbacks can make them altogether impractical [1,2,3,4,5,8]. In order to reduce the weight and energy consumption of such systems, they have been coupled with superhydrophobic surfaces [9,10]. These hydrophobic surfaces are useful, as they repel the impingement of supercooled water droplets, which is the primary cause of icing in many environmental applications [2,8]. One example of such systems is the hybrid Ice Coating and Electrothermal Heating Wing Ice Protection System (ICE-WIPS), which utilizes an internal heating element beneath a superhydrophobic surface, and reduces the power consumption by up to 70% as compared with other ice prevention systems [7].

Further energy savings can be achieved through the increased absorption of solar energy. A substantial number of superhydrophobic coatings, especially those used in de-icing systems, reflect most radiation in the visible range [11,12]. An increase in solar radiation absorbed would lead to a corresponding decrease in the energy required for ice removal. In this work, a coating was created which increased the absorption of solar radiation in the visible range, without sacrificing hydrophobic performance. One such polymer, polytetrafluoroethylene (PTFE), has been shown to be effective in de-icing systems, and demonstrates versatility in numerous conditions [13,14,15]. Previous studies have shown that modification of PTFE coating by additional molecules does not seriously impact the properties of the coating [14,15,16]. Graphite microparticles, due to their high absorptivity of radiation in the visible range as well as hydrophobic tendencies [17,18], are an effective additive to create an improved de-icing coating, with increased solar absorption coupled with super hydrophobicity. The characterization of the coating was carried out in three ways. To demonstrate substantial increase in the absorption of solar radiation, which in turn corresponds to heat generated on the surface of the coating, the coating was tested in a spectrometer. In order to verify that hydrophobic performance had not been impacted, contact and roll-off angle tests were performed. These results were compared with an unmodified superhydrophobic PTFE coating. Lastly, in order to demonstrate improved de-icing performance in environmental conditions, the coating was tested in an icing wind tunnel, where it was coated in ice, and then exposed to external radiation to simulate artificial sunlight.

## 2. Method and Experiment

In this work, a superhydrophobic coating was created through modification of an existing PTFE coating [19]. The components of the polymer were PTFE nanoparticles from Millipore Sigma binding and hardening agents from ShinEtsu, and a dispersant from 3M. The coating was modified by adding graphite microparticles at a mass ratio of 1:4 to PTFE. The ingredients of both the PTFE coating and the graphite coating are shown in Table 1.

The ingredients of the coating were mixed together and sonicated to ensure even distribution throughout the mixture. The mixture was then sprayed onto bare aluminum samples which had been sanded to remove the oxide layer. Point measurements of the coating thicknesses were taken at 5 mm increments on the aluminum samples using an Oxford Instruments thickness gauge from Abingdon, England. The average thickness for both coatings was 11.2 μm. The minimum coating thickness was 10 μm and the maximum was 13 μm.

### 2.1. Spectrometer Testing

The absorptivity of the graphite coating was analyzed using a HORIBA FluoroMax-4 spectrometer from Kyoto, Japan. 5 cm × 5 cm aluminum samples were spray coated and allowed to dry at 25 °C for 24 h. The samples were then analyzed with a synchronous test which directly measured the reflectance of the sample from 350 nm to 1150 nm, which encompasses the entire visible range, as well as a large portion of the infrared range where solar irradiance is high. This test was repeated 10 times for 3 aluminum samples in order to reduce uncertainty in the measurement. The local material property absorptivity could be used to calculate an overall absorptivity for the coating when exposed to solar radiation using Equation (1):

(1) $$\alpha_{overall}\approx \frac{\int^{\;}\alpha \left(\lambda \right)℘\left(\lambda \right)d\lambda }{\int^{\;}℘\left(\lambda \right)d\lambda }$$

where $\alpha_{overall}$ is the total absorptivity of the coating when exposed to a specific radiation source, α is the absorptivity of the coating as a function of wavelength, and ℘ is the radiative power emitted by a radiation source as a function of wavelength. The value of ℘(λ) can be estimated by approximating the light source as a black body at a given temperature. For solar radiation, the black body temperature was assumed to be 5800 K. The steps for calculating $\alpha_{overall}$ are shown in Appendix A. The overall absorptivity is related to total irradiative heat flux to a surface shown in Equation (2), where $\dot{q}$ is the heat flux to a surface, σ is the Stefan-Boltzmann constant, and *T* is the temperature of the irradiation source.

(2) $$\dot{q}=\sigma \alpha_{overall}T^{4}$$

Neither σ nor *T* depend on material properties, so the ratio of *α_overall_* is equal to the ratio of the irradiative heat flux to a give surface. This relationship is shown in Equation (3).

(3) $$\frac{{\dot{q}}_{Graphite}}{{\dot{q}}_{PTFE}}=\frac{\alpha_{Graphite}}{\alpha_{PTFE}}$$

### 2.2. Contact and Roll-Off Angle Tests

**Figure 1:**
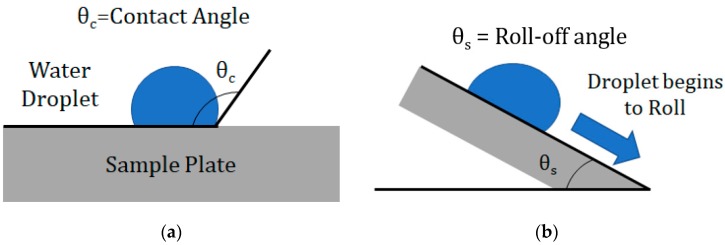
(**a**) Contact angle schematic; (**b**) roll-off angle schematic.

Due to various static and dynamic phenomena which impact hydrophobicity, characterization had to be done through analysis of both roll-off and contact angles [20]. Both of these angles were measured using a 10 μL droplet of pure water. The contact angle, which is a static characterization, uses the angle that the droplet makes with the coated surface as an indication of the wettability of that surface. An image-based angle determination was made using computer image analysis. A higher contact angle indicates increased hydrophobicity. A schematic of the contact angle is shown in Figure 1. 

The roll-off angle is a dynamic characterization which measures the angle to which a surface must be tilted for a droplet to roll. It is an indicator of the minimum force required to remove a droplet from the surface. In the characterization, the sample plate was rotated in increments of 1 degree until the droplet began to roll. A lower roll-off angle indicates increased hydrophobicity. A schematic of the roll-off angle is shown in Figure 1b. Each test was performed 10 times for 3 aluminum samples to minimize uncertainty in the measurement. 

The contact and roll-off angle tests were performed at 20 °C. Because both contact and roll-off angle tests were used to measure hydrophobic performance, the droplets did not need to be supercooled, as Morita et al. have demonstrated that temperature has negligible impact on the hydrophobicity of a surface [19]. For water at the length scale used, droplet size has minimal impact on contact angle [21].

### 2.3. Icing Wind Tunnel Tests

**Figure 2:**
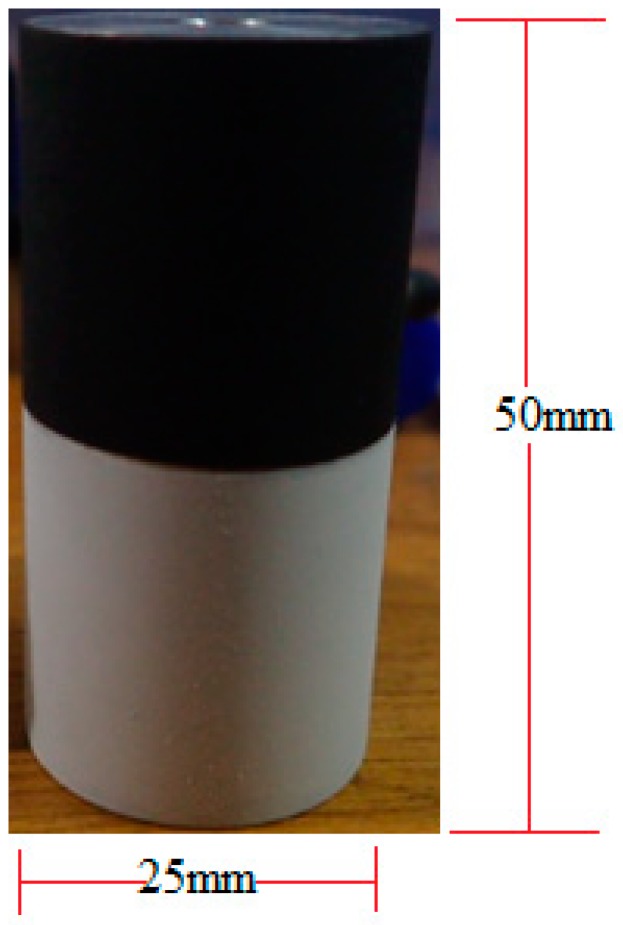
Cylinder coated with polytetrafluoroethylene (PTFE) coating (bottom) and graphite coating (top).

Icing wind tunnel tests were run in order to test the performance of the coatings in environmental icing conditions. Coated cylinders were placed in the Kanagawa Institute of Technology icing wind tunnel. These cylinders were 25 mm in diameter and 50 mm long. An example of a model with both coatings can be seen in Figure 2.

Each coated cylinder was rotated at 15 rpm in supercooled airflow in order to create a uniform sheet of ice over the coated surface. The flow exposure lasted for 3 min, and the parameters of the flow, including temperature, flow velocity, relative humidity, Liquid Water Content (LWC), and Median Volume Diameter (MVD) were varied to generate different forms of environmental icing. LWC is a measure of the mass of water present in the airflow, and MVD is the median diameter of the liquid droplets suspended in the airflow. Both of these values were adjusted by varying the nozzle water and air pressure. The nozzle pressures were calibrated to produce specific MVD and LWC values before testing using a multiple rotating cylinders calibration method [22]. The various test flow conditions are shown in Table 2.

**Figure 3:**
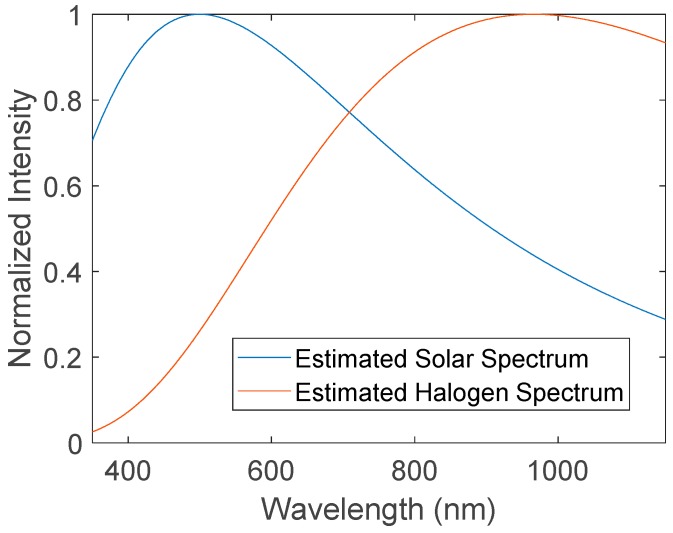
Halogen and solar intensity as a function of wavelength.

To be certain that the ice accumulation was constant across different tests, the models were weighed before and after being coated in ice. After weighing, the coated samples were returned to the wind tunnel and exposed to a 50 W MR16 halogen lamp with a color temperature of 3000 K at a distance of 10 cm to simulate solar radiation. The spectrum of the halogen irradiation as compared to solar irradiation is shown in Figure 3.

Intensity from the halogen bulb peaks much later than solar radiation. However, the flatter shape of the halogen spectrum means that a substantial portion of the irradiative power is present in the visible range, where the greatest difference between the absorption spectra of the coatings is expected. Therefore, the halogen light source is able to demonstrate a difference in overall absorptivity, and thus heat absorption, between the two coatings. 

**Figure 4:**
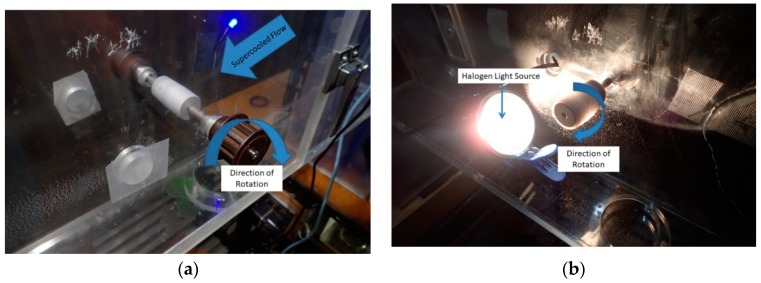
(**a**) Coating during supercooled airflow exposure and (**b**) radiation exposure.

The cylinders were again rotated at 15 rpm to ensure an even exposure to radiation. The amount of time required for ice to be fully removed from the surface was measured, and then divided by the measured ice mass. The setup of both the flow and radiation portions of the test are shown in Figure 4. 

## 3. Results

### 3.1. Energy Absorption Coefficient 

**Figure 5:**
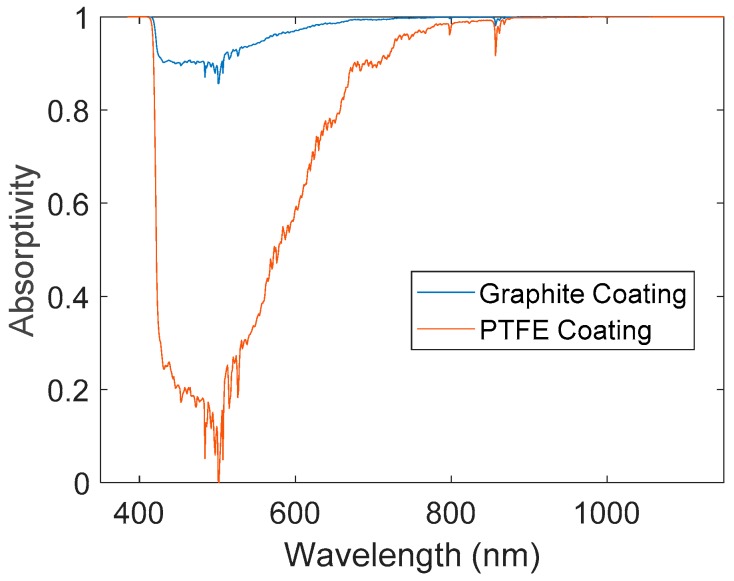
Local absorptivity as a function of wavelength.

The data collected from the spectrometer is shown in Figure 5. Over the entire visible range, the absorptivity of the graphite coating is significantly higher than that of the PTFE coating. At 501 nm, which is near the peak intensity of solar irradiation, the PTFE coating shows a minimum absorptivity of 6.35 × 10^−3^, while the graphite coating has a local absorptivity of 0.857. As described in Equation (1), the spectra were multiplied by the solar and halogen irradiation intensity and integrated to find an overall absorptivity coefficient. These coefficients are shown in Table 3. 

The overall absorptivity coefficient for the graphite coating was 35% greater than that of the PTFE coating under exposure to typical solar radiation, and 9.3% greater under exposure to the halogen light source. These increases in absorptivity correspond to identical increases in heat absorbed due to irradiation. 

### 3.2. Hydrophobic Performance 

The average contact and roll-off angle measurements for both coatings, compared to a perfectly hydrophobic surface are shown in Table 4.

The coatings show identical hydrophobic performance within uncertainty. 

### 3.3. Icing Wind Tunnel Results

**Figure 6:**
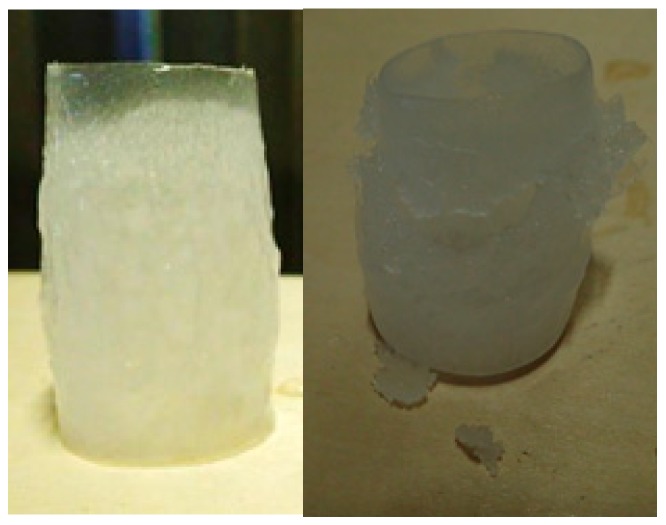
Removed ice sheets from graphite coating (left) and PTFE coating (right).

Based on qualitative observations during the de-icing process, the ice sheet on the graphite coating melted at the base layer of the coating, and was removed in a single, uniform sheet. However, the ice sheet on the PTFE coating melted more from the outside, and in pieces, requiring substantially longer for the ice to be completely removed. The ice sheets after removal from the models are shown in Figure 6. 

The accumulated ice mass on the surface of the models after exposure to supercooled airflow is shown in Table 5. As shown, the ice accumulation was very similar for each coating, but varied dramatically with ambient temperature.

**Figure 7:**
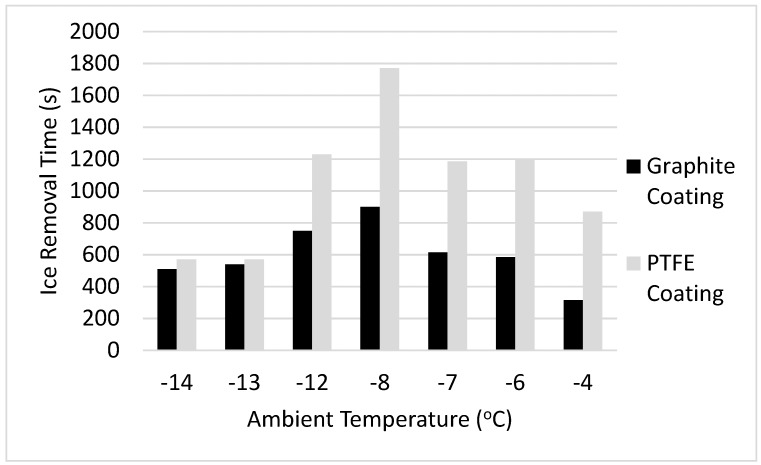
Ice removal time vs. test section temperature.

**Figure 8:**
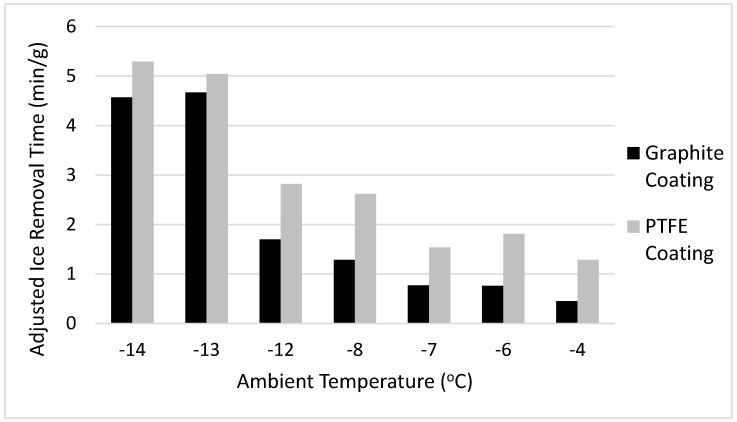
Adjusted ice removal time vs. test section temperature.

The time required for complete ice removal is shown in Figure 7, and the time adjusted by ice mass is shown in Figure 8.

As shown, the time required for ice removal was substantially lower for the graphite coating as compared to the PTFE coating. In glaze ice conditions, or temperatures between −12 °C and −4 °C, the ice on the graphite was removed more than 50% faster when compared to the PTFE coating. In rime ice conditions, or temperatures between −14 °C and −13 °C, the ice removal time for the graphite coating was an 8.5% improvement over the PTFE coating. Such a sharp change in improvement time is due to the different opacities of the ice, which changes the spectrum received at the coating layer, the overall absorptivity coefficient, and the heat absorbed by the coating. 

## 4. Conclusions and Discussion

The improved absorptivity of the graphite coating is clearly evident in the spectrometer analysis, and the highest improvement in absorptivity over an unmodified PTFE coating occurs at a wavelength of 501 nm, near the peak intensity of solar irradiation spectrum [23]. The increase in the absorptivity coefficient is directly related to the radiation absorbed and heat captured by the graphite coating, which is described in Equations (2) and (3). An increase in heat captured passively by the coating would directly reduce the amount of energy required from an active heating unit. 

The hydrophobic characterization through the contact and roll-off angle tests demonstrate no decrease in hydrophobic performance in the graphite coating, which maintained superhydrophobicity characteristic of a PTFE coating even after modification with graphite. These roll-off and contact angle tests indicate identical wettability and surface energies, within uncertainty. The graphite could be added to the coating without interfering with the PTFE microstructure, which is required for the hydrophobicity of the coating.

The icing wind tunnel tests demonstrated the feasibility of the graphite coating in environmental icing conditions, and both a qualitative and quantitative observation of de-icing performance indicates improvement through the addition of graphite. Upon observing the ice sheets after they had been removed from the coating surface, it can be concluded that the graphite coating promoted substantially more melting at the base layer of the ice, which is what allowed the ice sheet to be removed in a single piece. This increase in base layer melting is due to higher absorption of radiation, which translates to higher heat generation at the coating surface. Analysis of the ice removal times shows that the graphite coating removed ice more than 50% faster in glaze ice conditions, and 8.5% faster in rime ice conditions. Rime ice, due to its opacity, limits the spectrum which penetrates to the base layer of the ice, which reduces the relative effectiveness of the coating. However, in every performance metric over a variety of test conditions, the graphite coating showed similar performance to, or an improvement over, the standard PTFE coating. 

## Figures and Tables

**Table 1:** Coating ingredients for 5 cm × 5 cm sample plate.

PTFE Coating	Graphite Coating
2 g PTFE nanoparticles (1 μm diameter)	1 g PTFE nanoparticles (1 μm diameter)
60 μL KP-109 Silicon	60 μL KP-109 Silicon
10 mL Novec 7300 Engineered Fluid	10 mL Novec 7300 Engineered Fluid
1 mL KR-400 Hardener	1 mL KR-400 Hardener
-	25 g graphite microparticles (<20 μm diameter)

**Table 2:** Icing flow conditions.

Condition	Rime Ice	Glaze Ice
Temperature Range (°C)	[−14, −13]	[−12, −4]
Velocity (m/s)	10	40, 80
Humidity (%)	[45,50]	[40,45,55]
LWC (g/m^3^)	1.035	1.035
MVD (μm)	26.6	95.5
Air Volume (L/min)	20	20
Water Volume (mL/min)	20	15

**Table 3:** Coefficients of solar absorption for various coating types.

Coated Surface	α_overall_ (solar)	α_overall_ (halogen)
PTFE coating	0.719	0.907
Graphite coating	0.971	0.992

**Table 4:** Roll-off and contact angle measurements.

-	PTFE Coating	Graphite Coating	Ideal
Contact Angle (^o^)	157 ± 4	155 ± 4	180
Roll-off Angle (^o^)	4 ± 1.3	4 ± 1.2	0

**Table 5:** Ice accumulation for various ambient temperatures.

Ambient Temperature (°C)	Graphite Coating Ice Accumulation (g)	PTFE Coating Ice Accumulation (g)
−4	11.6562	11.2918
−6	12.865	12.0634
−7	13.3347	12.8716
−8	11.6562	11.2718
−12	7.3577	7.2755
−13	1.92785	1.88485
−14	1.8611	1.7961

## Appendix A

Calculation of $\alpha_{overall}\approx \frac{\int^{\;}\alpha \left(\lambda \right)℘\left(\lambda \right)d\lambda }{\int^{\;}℘\left(\lambda \right)d\lambda }$:

The value for ℘(λ) for both the solar and halogen spectrums was estimated using Planck’s Law, shown in Equation (A1):

(A1) $$℘\left(\lambda \right)=\frac{8\pi hc}{\lambda^{5}}\frac{1}{\exp\left(\frac{hc}{kT\lambda }\right)-1}$$

where ℘ is local irradiative power, λ is wavelength, *h* is the Planck Constant, *c* is the speed of light, *T* is the temperature of the black body, and *k* is the Boltzmann constant. This curve was then discretized and multiplied by the data collected from the spectrometer for α(λ). The discretized functions were then integrated using a trapezoidal integration method to produce a value for $\alpha_{overall}$.
